# Mild inhibition of mitochondrial complex I improves glucose metabolism and energy homeostasis in a cellular model of Alzheimer’s disease

**DOI:** 10.1002/alz.085373

**Published:** 2025-01-03

**Authors:** Thi Kim Oanh Nguyen, Sergey A Trushin, Mark Ostroot, Eugenia Trushina

**Affiliations:** ^1^ Department of Neurology, Mayo Clinic, Rochester, MN USA; ^2^ Department of Molecular Pharmacology and Experimental Therapeutics, Mayo Clinic, Rochester, MN USA

## Abstract

**Background:**

While disease‐modifying treatments that reduce Aβ have been recently approved by the FDA, the identification of novel therapeutic targets and strategies that target underlying mechanisms to delay the AD development are still needed. Abnormal brain energy homeostasis and mitochondria dysfunction are observed early in AD. Therefore, the development of treatments to restore these defects could be beneficial. We identified small molecule (code name CP2) as a mild and specific mitochondrial complex I (MCI) inhibitor. Application of CP2 improved brain energy homeostasis, restored synaptic and cognitive function in APP/PS1 and 3xTgAD mice. However, mechanistic relationship between MCI inhibition, glucose uptake/utilization and mitochondrial function remains to be determined.

**Method:**

Cellular energy metabolism was assessed in the neuroblastoma SH‐SY5Y cells that express either mutant human APP protein (APPswe) or empty vector (control) treated with vehicle or CP2. A Seahorse Extracellular Flux Analyzer was used to measure glycolysis, oxygen consumption rate, and fatty acids β‐oxidation (FAO). Flow cytometry allowed determining the translocation of glucose transporters to the cell surface. Changes in protein expression in response to treatment were assessed using Western Blot analysis. Changes in mitochondrial morphology were monitored using electron microscopy. The non‐radioactive Glucose Uptake‐Glo™ assay was utilized for measuring glucose uptake in cells. Metabolic flux analysis was done using ^13^C D‐Glucose stable isotope‐labeling.

**Results:**

APPswe cells have a significant decrease in glycolysis and spare respiratory capacity, an indicator of the mitochondrial ability to produce energy under stress conditions. These observations were consistent with decreased glucose uptake, which was compensated by an increased FAO to provide axillary fuel for ATP production. Mechanistically, at the concentrations relevant to *in vivo* treatment in APP/PS1 and 3xTgAD mice, acute CP2 treatment increased glucose uptake and utilization through the translocation of glucose transporters to the plasma membrane while prolong CP2 treatment activated metabolic sensors and mitochondrial morphofunctional pathways (e.g., fission, fusion, biogenesis, and turnover) consistent with the improved cellular bioenergetics in the AMPK‐dependent pathway.

**Conclusion:**

Data suggested that mild MCI inhibition activates multiple neuroprotective mechanisms improving cellular energy homeostasis and mitochondrial function *in vivo* and *in vitro*, representing promising strategy that target early AD mechanisms.

